# Knowledge, attitudes, perceptions, and practice toward seasonal influenza and its vaccine: A cross-sectional study from a country of conflict

**DOI:** 10.3389/fpubh.2023.1030391

**Published:** 2023-02-13

**Authors:** Wesam S. Ahmed, Rana Abu Farha, Abdulsalam M. Halboup, Arwa Alshargabi, Ahmed Al-mohamadi, Eman Y. Abu-rish, Mohammed Zawiah, Yousf K. Al-Ashbat, Sayida Al-Jamei

**Affiliations:** ^1^College of Health and Life Sciences, Hamad Bin Khalifa University, Qatar Foundation, Doha, Qatar; ^2^Faculty of Pharmacy, Applied Science Private University, Amman, Jordan; ^3^Department of Clinical Pharmacy and Pharmacy Practice, Faculty of Pharmacy, University of Science and Technology, Sana'a, Yemen; ^4^Department of Clinical Pharmacy, School of Pharmaceutical Sciences, Universiti Sains Malaysia, Penang, Malaysia; ^5^Faculty of Dentistry, Saba University, Sana'a, Yemen; ^6^Department of Biopharmaceutics and Clinical Pharmacy, School of Pharmacy, The University of Jordan, Amman, Jordan; ^7^Department of Pharmacy Practice, College of Clinical Pharmacy, Hodeidah University, Al Hodeidah, Yemen; ^8^Department of Clinical Pharmacy and Therapeutics, Faculty of Pharmacy, Al-Razi University, Sana'a, Yemen

**Keywords:** seasonal influenza, Yemen, vaccination coverage, knowledge, barriers, influenza vaccine, motivators, vaccine uptake

## Abstract

**Background:**

The seasonal influenza vaccine is an important preventive measure against influenza and its associated complications. In Yemen, there is no seasonal influenza vaccination policy, and the influenza vaccine is excluded from the national immunization program. Data on vaccination coverage remain scarce with no previous surveillance programs or awareness campaigns implemented in the country. The current study aims to assess the awareness, knowledge, and attitudes of the public in Yemen toward seasonal influenza and their motivators and perceived barriers to receiving its vaccine.

**Methods:**

A cross-sectional survey was carried out using a self-administered questionnaire that was distributed to eligible participants using convenience sampling.

**Results:**

A total of 1,396 participants completed the questionnaire. The respondents showed a median knowledge score of influenza of 11.0/15.0, and most of them (70%) were able to recognize its modes of transmission. However, only 11.3% of the participants reported receiving the seasonal influenza vaccine. Physicians were the respondents' most preferred information source for influenza (35.2%), and their recommendation (44.3%) was the most cited reason for taking its vaccine. On the contrary, not knowing about the vaccine's availability (50.1%), concerns regarding the safety of the vaccine (17%), and not considering influenza as a threat (15.9%) were the main reported barriers to getting vaccinated.

**Conclusion:**

The current study showed a low uptake of influenza vaccines in Yemen. The physician's role in promoting influenza vaccination seems to be essential. Extensive and sustained awareness campaigns would likely increase the awareness of influenza and remove misconceptions and negative attitudes toward its vaccine. Equitable access to the vaccine can be promoted by providing it free of charge to the public.

## 1. Introduction

Seasonal influenza is an acute infection of the respiratory tract caused by influenza virus type A, B, or C (1). Influenza virus types A, B, and C are known to infect humans, while type D is believed to infect cattle (2, 3). Type A influenza virus is subdivided into several serotypes based on the viral hemagglutinin (H) and neuraminidase (N) surface proteins. Some of these serotypes were responsible for outbreaks throughout recent history (4). Most notoriously are the 1918 Spanish flu (H1N1), the 1857 Asian flu (H2N2), the 1068 Hong Kong flu (H3N2) (5), and the 2009 Swine flu outbreak (H1N1) (6). Other viral respiratory pandemics in the last couple of decades include the 2002–2004 severe acute respiratory syndrome (SARS) outbreak caused by the newly identified—at the time—SARS coronavirus (SARS-CoV) (7), and the ongoing coronavirus 2019 (COVID-19) pandemic caused by a related coronavirus strain, SARS-CoV-2 (8–10).

Uncomplicated seasonal influenza is manifested by a combination of symptoms, most commonly headache, cough, sore throat, runny nose, fatigue, muscle pain, and fever (11, 12). Complications of influenza include pneumonia, sinus and ear infections, and worsening of existing chronic medical conditions such as asthma, diabetes, and heart failure (13). According to the World Health Organization (WHO), seasonal influenza afflicts one billion individuals worldwide annually resulting in 3–5 million severe illness cases and between 290,000 and 650,000 influenza-associated respiratory mortality (14). Moreover, the disease poses an additional load on the healthcare system and increases the economic burden as a result of work absenteeism and loss of productivity (15). High-risk groups of influenza complications include immunocompromised individuals, patients with chronic conditions, pregnant females, adults older than 65 years of age, and children younger than 5 years old with those younger than 2 years of age being at even higher risk of influenza complications (16, 17). Healthcare workers (HCWs) are also considered an at-risk group (12).

The seasonal influenza vaccine (flu vaccine) is an important preventive measure against influenza and its associated complications (18). The vaccine protects against 3 or 4 influenza viruses that are expected to circulate in the upcoming flu season (18). The directors of the WHO collaborating centers, laboratories, and academies recommend the composition of the flu vaccine based on surveillance and clinical studies (19). The Centers for Disease Control and Prevention (CDC) recommend all individuals aged ≥6 months take the vaccine (18, 20, 21) by the end of October each year (21). Priority is given to healthcare workers and other high-risk groups (16, 22, 23). The flu vaccine provides several benefits including protection against flu infection (24–26), severity (27), and hospitalization (28), especially in high-risk groups (29–33).

In Yemen, little is known about the public's knowledge of influenza, and their attitudes and practice toward its vaccine. According to the WHO Regional Office of the Eastern Mediterranean region, the country has neither a seasonal influenza vaccination policy for the general public or subgroups nor does it include the influenza vaccine in its national immunization program (34). Despite increased recommendations on the value of influenza vaccination, data on the burden of influenza and vaccination coverage in the country remain scarce (34). In addition to COVID-19 (35), the country is simultaneously struck by three other fever-causing infectious diseases: dengue fever, chikungunya fever, and malaria (36–38). Thus, it is imperative to address these severe infections and complement this with the implementation of equitable influenza vaccine programs to reduce the overall disease burden and exhaustion of medical resources which are already scarce in the country as a result of the ongoing war. No previous surveillance programs or awareness campaigns that cover influenza and its vaccine have been implemented in the country. Therefore, the aim of the current study is to assess knowledge, attitudes, and practices toward influenza and its vaccine among the public in Yemen and to understand the main determinants of vaccine acceptance which could be a critical step toward future planning of national influenza campaigns and in implementing national influenza vaccination policy to improve vaccination coverage in the country. To the best of our knowledge, this is the first study to address this topic in the country.

## 2. Methods

### 2.1. Study design and participants

A questionnaire survey was distributed in public places in Sana'a city to eligible participants over the period of March 2019 to February 2020 using a convenience sampling approach. Potential participants were approached on the streets by experienced interviewers and were invited to participate in the survey. The face-to-face sampling was then discontinued due to the COVID-19 outbreak. This was followed by an online recruitment phase where the questionnaire survey was deployed online using social media platforms (WhatsApp and Facebook) between the 5th of July and the 25th of October 2020. The online sampling included participants from different cities. The survey was directed to the public in Yemen who are aged 18 years or older, are competent, and can read and understand the Arabic language. Consent was obtained orally for the face-to-face sampling. For online recruitment, a consent statement was included at the beginning of the questionnaire as well as in the recruitment invite that was shared through social media platforms.

The questionnaire was adopted from a previously validated questionnaire (39) with few modifications to be applicable to the public in Yemen. The questionnaire was distributed in Arabic since it is the native language of the country. Several response formats were utilized in the questionnaire, including multiple choice, “Yes,” “No,” or “I am not sure,” multiple check box, the Likert scale, and open-ended items. The survey consisted of four sections. The first section solicited sociodemographic information from respondents, including age, gender, marital status, education level, and whether they have ever taken the flu vaccine before. Demographic questions also focused on whether the respondents are working in the medical field, have any chronic medical conditions, or are medically insured. The following section of the questionnaire was designed to assess participants' knowledge of influenza, its modes of transmission, and its preventive measures. The third section assessed the source of knowledge participants used to gain information about influenza and its vaccine. The last section of the questionnaire was designed to determine participants' motivating factors and barriers toward taking the influenza vaccine. The study was verified by the ethics committee of the Scientific Research Center of Yemen University (Ref #: ERC/2018/123).

### 2.2. Data analysis and figure preparation

Data were analyzed using the statistical package for social science (SPSS) version 22 (SPSS Inc., Chicago, IL, USA). The descriptive analysis was executed using the median and interquartile range for continuous variables and frequency (percentage) for qualitative variables. Checking for data normality was carried out using the Shapiro–Wilk test (with *P* > 0.05 indicating a normally distributed continuous variable). To assess the respondents' level of knowledge of influenza, a score of 1 was given to each correct answer to the 15 questions exploring general knowledge, mode of transmission, and preventive measures. A score of 0 was given for wrong answers. The total score in this construct ranged from 0 to 15. Screening for factors affecting participants' previous uptake of the seasonal influenza vaccine was carried out using univariate and multivariate logistic regression. Following univariate logistic regression analysis, any variable found to be significant on the single predictor level (*P*-value < 0.25) was entered into the multivariate logistic regression analysis to explore the factors that were significantly and independently associated with participants' previous uptake of the seasonal influenza vaccine. Odds ratios were calculated to measure the effect of each predictor on the practice of seasonal influenza vaccine uptake. Variables were selected after checking their multicollinearity, where tolerance values >0.1 and variance inflation factor (VIF) values < 10 were checked to indicate the absence of multicollinearity between the independent variables in regression analysis. A *p*-value of ≤0.05 was considered to be statistically significant. Figures were prepared using Microsoft Excel 13.

## 3. Results

### 3.1. Demographics

The demographic analysis is reported in Table 1. A total of 1,396 participants completed the questionnaire (472 on-site and 924 online). The response rate for the on-site sampling was 61%. The completion rate for the on-site and online sampling was 72%. Approximately 41% (*n* = 575) were aged between 18 and 24 years of age, and men were overrepresented (63.0%, *n* = 879). Most participants had a diploma or a higher education degree (80.5%, *n* = 1,124), and approximately one-third were from the medical field (36.9%, *n* = 515). Many participants were from big cities such as Sana'a (34%, *n* = 476), Taiz (23.4%, *n* = 326), and Ibb (12%, *n* = 172). A minority were smokers (12.2%, *n* = 172), and 12.2% (*n* = 170) reported having a chronic medical condition. Most participants were not medically insured (75.4%, *n* = 1,053). In addition, 11.3% (*n* = 158) stated that they had received the seasonal influenza vaccine before (Table 1).

**Table 1:** Socio-demographic characteristics of the study sample (*n* = 1,396).

**Parameter**	***n* (%)**
**Age (years)**	
18–24	575 (41.2)
25–35	496 (35.5)
≥35	325 (23.3)
**Gender**	
Males	879 (63.0)
Females	517 (37.0)
**Marital status**	
Married	630 (45.1)
Others	766 (54.9)
**Education level**	
School level	255 (18.3)
Diploma	118 (8.5)
Undergraduate degree	874 (62.6)
Post-graduate degree	132 (9.5)
Missing data	17 (1.2)
**Governorate**	
Sana'a	476 (34.1)
Taiz	326 (23.4)
Ibb	172 (12.3)
Hodidah	58 (4.2)
Hajah	58 (4.2)
Others	306 (21.9)
**Are you from the medical field?**	
No	881 (63.1)
Yes	515 (36.9)
**Do you have any chronic medical conditions?**	
No	1,225 (87.8)
Yes	171 (12.2)
**Do you have medical insurance?**	
No	1,053 (75.4)
Yes	343 (24.6)
**Smoking status**	
Non-smoker/ex-smoker	1,225 (87.8)
Current smoker	171 (12.2)
**Have you ever had the seasonal influenza vaccine?**	
No	1,233 (88.3)
Yes	158 (11.3)
Missing data	5 (0.4)

### 3.2. Participants' knowledge

**Figure 1:**
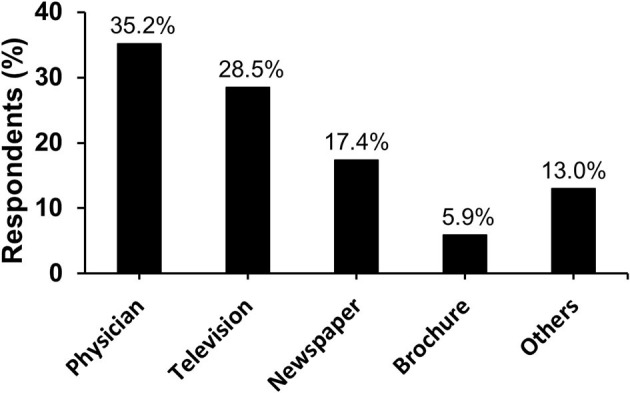
Information sources that the participants utilized to gain knowledge about influenza (*n* = 1,396).

Participants' knowledge of influenza was assessed and is reported in Table 2. Participants showed a median knowledge score of 11.0 out of 15.0. Most participants correctly identified the definition of influenza (82.7%, *n* = 1,155), its risk factors such as comorbid chronic diseases (75.8%, *n* = 1,058) and age ≥65 years and ≤5 years (70.9%, *n* = 990). However, only 21.5% (*n* = 300) were aware that not every H1N1-infected person will experience complications that need hospitalization and 16.8% (*n* = 234) thought that cures are available to treat complicated cases of influenza. Considering modes of transmission and prevention, more than 77% were able to recognize the different modes of influenza virus transmission, and the majority believed that avoiding crowded places helps prevent the transmission (90.0%, *n* = 1,257), although influenza vaccine was the least recognized preventive measure (62.5%, *n* = 872). Sources of information are illustrated in Figure 1.

**Table 2:** Participants' knowledge of influenza (*n* = 1,396).

**Item**	**Correctly answered**
	***n*** **(%)**^†^
**General knowledge**	
Influenza is a contagious respiratory infection that is caused by respiratory viruses such as H1N1, and might cause an illness ranging from mild symptoms to serious pneumonia^‡^	1,155 (82.7)
Swine influenza is a type of influenza caused by H1N1 virus^‡^	922 (66.0)
All H1N1 infected individuals will experience complications that need hospitalization^§^	300 (21.5)
All H1N1 infected individuals will die as a result of the infection^§^	750 (53.7)
Cures are available for the treatment of serious cases of influenza^§^	234 (16.8)
People with chronic conditions (such as asthma, COPD, heart diseases and/or diabetes) have a higher risk of developing serious influenza complications^‡^	1,058 (75.8)
Elderly (≥65 years old) and children (≤5 years old) have a higher risk of developing serious influenza complications^‡^	990 (70.9)
**Mode of transmission**	
Influenza can spread through unprotected contact with respiratory droplets of infected individuals^‡^	1,162 (83.2)
Influenza can spread through mouth droplets of infected individuals when they cough, sneeze or talk^‡^	1,258 (90.1)
Influenza can spread through touching mouth or nose after contact with contaminated objects^‡^	1,089 (78.0)
**Preventative measures**	
Wearing mask can limit the spread of influenza^‡^	1,220 (87.4)
Covering your nose or mouth when sneezing can limit the spread of influenza^‡^	1,245 (89.2)
Washing hands with water and soap after coughing or sneezing can limit the spread of influenza^‡^	1,245 (89.2)
Avoiding crowded places helps to limit the spread of influenza^‡^	1,257 (90.0)
Getting vaccinated can limit the spread of influenza^‡^	872 (62.5)
Knowledge score [median (IQR)]	11.0 (3.0)

† Participants can select more than one choice, so total percentage may exceed 100.

‡ The correct answer to these statements is “Yes.”

§ The correct answer to these statements is “No.”

COPD, chronic obstructive pulmonary disease, IQR, interquartile range.

### 3.3. Motivators, barriers, and factors influencing vaccine uptake

Participants who reported receiving the flu vaccine cited the motivating factors that contributed to their vaccine acceptance (Table 3). Compliance with the physician's recommendation (44.3%, *n* = 70) was the most cited motivator for taking the flu vaccine, followed by fear of catching H1N1 influenza (29.7%, *n* = 47) and preventing disease transmission to family members (16.5%, *n* = 26).

**Table 3:** Factors affecting participants' practice toward seasonal influenza vaccine (*n* = 1,396).

**Factor**	**Participants agreed, *n* (%)^†^**
**Reasons for getting vaccinated [reported by participants who had ever received the vaccine (*****n*** **= 158)]**	
Compliance with physician's recommendation	70 (44.3)
Fear from catching H1N1 influenza	47 (29.7)
Worries about becoming severely ill following influenza infection	18 (11.4)
To prevent disease transmission to family members	26 (16.5)
Having a chronic medical condition	4 (2.5)
**Reasons for not getting vaccinated [reported by participants who had never received the vaccine (*****n*** **= 1,233)]**	
Not considering influenza as a threat	196 (15.9)
Doubts regarding the vaccine's efficacy	121 (9.8)
Doubts regarding the vaccine's safety	210 (17.0)
Time constraints	55 (4.5)
Unaware of vaccine availability	618 (50.1)
Cost of the vaccine	50 (4.1)

† Participants can select more than one choice, so total percentage may exceed 100%.

On the contrary, participants who had never been vaccinated reported being unaware of vaccine availability (50.1%, *n* = 618) as the most common barrier for not getting vaccinated, followed by safety concerns regarding the vaccine (17.0%, *n* = 210) and not considering influenza as a threat (15.9%, *n* = 196) (Table 3).

In addition, participants reported factors that would encourage them to get vaccinated in the future (Table 4). Approximately two-thirds agreed or strongly agreed to take the vaccine if it was recommended by their physician (67.4%, *n* = 941) or if the vaccine was better validated for safety and efficacy (66.4%, *n* = 918).

**Table 4:** Factors that will encourage participants to get vaccinated in future (*n* = 1,396).

**Factor**	**Strongly agreed/agreed (%)^†^**	**Missing data**
The vaccine is recommended by the physician	941 (67.4)	5 (0.4)
The vaccine is more validated for safety and efficacy	918 (66.4)	5 (0.4)
Vaccine uptake is encouraged by the government	835 (59.8)	5 (0.4)
The vaccine is offered free of charge by the government	891(63.8)	5 (0.4)

† Participants can select more than one choice, so total percentage may exceed 100%.

### 3.4. Predictors for low vaccination uptake

The results from univariate logistic regression on a single predictor level (*P*-value < 0.25) revealed that the practice toward seasonal influenza uptake was significantly less frequent among participants who are women, have a diploma or higher education degree, work in the medical field, and are older in age. These factors were further analyzed through multivariate linear regression analysis (backward method) to explore the factors that were significantly and independently associated with low prior vaccine uptake (*P* ≤ 0.05 with OR < 1). Two factor fulfilled the criteria, these are older age (OR = 0.967, *P* = 0.040) and being from the medical field (OR = 0.686, *P* = 0.044) (Table 5). The model fit was found to be significant with χ^2^ (df = 4) = 17.491 at *P* = 0.002, which indicated that our full model predicts significantly better or more accurately than the null model.

**Table 5:** Assessment of factors affecting participants' practice toward seasonal influenza vaccine uptake (*n* = 1,396).

**Parameter**	**Previous vaccine uptake [0: No, 1: Yes]**			
	**OR**	***P*** **-value** ^†^	**OR**	***P*** **-value** ^‡^
Age (years)	0.967	0.005^§^	0.976	0.040^*^
**Gender**				
Male	Reference			
Female	0.697	0.049^§^	0.709	0.064
**Marital status**				
Married	Reference			
None-married (single, widowed, or divorced)	1.134	0.462	–	–
**Educational level**				
School or lower	Reference			
Diploma of higher	0.768	0.199^§^	0.693	0.104
**Are you from the medical field?**				
No	Reference			
Yes	0.665	0.017^§^	0.686	0.044^*^
**Do you have any chronic medications?**				
No	Reference			
Yes	0.938	0.726	–	–
**Do you have medical insurance?**				
No	Reference			
Yes	1.006	0.976	–	–
**Smoking status**				
Non-smoker/ex-smoker	Reference			
Current smoker	0.908	0.714	–	–
Knowledge score	1.038	0.270	–	–

† Using simple logistic regression.

‡ Using multiple logistic regression.

§ Eligible for entry in multiple logistic regression.

* Significant at 0.05 significance level.

## 4. Discussion

Influenza is an overlooked contributor to morbidity and mortality (40–42). Several studies assessed knowledge about influenza and attitudes toward its vaccine among healthcare professionals (43–50). However, studies to assess the same among the public are generally scarce (46, 51–53), especially in a low-income, war-torn developing country such as Yemen. This is the first large-scale study in the country to assess the public's knowledge, attitudes, and perceptions of influenza and its vaccine, and their motivating factors and perceived barriers to vaccine acceptance. Findings from this study will inform future national influenza vaccination regimes and assess improving vaccination coverage in the country.

Overall, the results from the current study showed an acceptable median score of knowledge of influenza among the participants, but some major gaps in knowledge were identified. Physicians were the main source that participants sought to gain information about influenza. Only a minority of participants had ever received the influenza vaccine. Lack of awareness of vaccine availability in the country was identified as the main barrier toward vaccine uptake, while physicians' recommendation to take the vaccine was cited as the prime motivator behind receiving the vaccine. Older age and being a healthcare worker were identified as predictors for low prior vaccine acceptance.

Assessing knowledge about influenza, its modes of transmission, and its preventive measures revealed an acceptable median knowledge score but with critical knowledge gaps that were mostly related to H1N1-associated infection. The median knowledge score would seem to reflect the education level of the participants as most participants reported receiving a higher education degree. The exaggeration of the H1N1 fatality risk reported by the participants in the current study is in disagreement with a former study from China where only a minority believed that H1N1 has a high fatality rate (54). On the contrary, although the majority correctly identified all influenza preventive measures, the influenza vaccine was the least recognized preventive strategy. This is similar to other studies from Italy and Jordan, where most participants did not recognize the vaccine as a major preventive measure to control influenza transmission (39, 46). These findings highlight the need for educational campaigns in the country to raise public awareness of influenza, especially H1N1, and the role of the flu vaccine in preventing the spread of infection.

Assessments of major sources of information that participants used to gain medical information about influenza revealed that physicians were the main source followed by television and newspapers. As a result, these information sources can be utilized in the future to enhance awareness of influenza and its vaccine among the public in Yemen. This is different from a study in Saudi Arabia where less than one-fourth of participants reported receiving information from a healthcare provider (55). In comparison, in a study from Jordan, a country that has the highest literacy rate in the Arab region (56, 57), newspapers were the major source of influenza information (39). In the United Kingdom, television and the Internet were the leading sources of knowledge about influenza (52). As such, it seems that education level and cultural differences between countries play a role in selecting a reliable source of information about influenza and its vaccine. Importantly, these findings suggest a higher trust in physicians' knowledge among the public in Yemen.

Regarding influenza vaccine uptake, although the respondents had a median knowledge score of 11/15, and most (62.5%) believed that the flu vaccine is an important preventive measure, the practice toward influenza vaccine uptake was relatively poor. Only a minority (11%) of respondents reported receiving the seasonal influenza vaccine previously. This low vaccine uptake may as well be an overestimation of the vaccination coverage for any given year, which has not been investigated in the current study. This is in agreement with the vaccination status reported in the MENA region according to the 7th Middle East and North Africa Influenza Stakeholder Network (MENA-ISN) report (58). As such, having knowledge about influenza and its vaccine does not seem to predict vaccination uptake. More evidence of this is that healthcare workers were less likely to receive the vaccine compared to those who were not enrolled in the medical field. Previous studies from the Arab MENA region such as Lebanon (44), Jordan (39), Saudi Arabia (45), Kuwait, Oman, and the United Arab Emirates (43) showed that individuals enrolled in a non-health related field had higher vaccination acceptance rate compared to those working in a medical field. This observation is not confined to the Arab MENA region but also extends globally (46, 51, 59). In a study from France, general practitioners who perceived the risk of influenza illness to outweigh the risk of its vaccination had a higher vaccination acceptance rate compared to those who did not perceive the same (51). Therefore, underestimating the risks and complications of influenza and overestimating the risks associated with its vaccine among individuals working in the medical field appears to be a major barrier to vaccination acceptance in this group. In addition to being from the medical field, the current study also identified older age as a predictor of low vaccine acceptance. Although older adults are at higher risk of influenza complications, this group is more hesitant about vaccine uptake. Similar findings were reported in other countries (60–64).

In addition to respondents' beliefs and knowledge, the current study assessed factors that contributed to prior vaccine acceptance or rejection. In agreement with some previous studies (39, 65, 66), physician recommendation to receive the vaccine was the main motivating factor for vaccine uptake. This, again, indicates trust in physicians' knowledge and recommendations among the respondents. Fear of catching an H1N1 infection was also reported as a major motivator to receiving the vaccine. On the contrary, not knowing about the availability of the vaccine in the country was regarded as the main barrier to getting vaccinated. Surprisingly, although Yemen is a low-income developing country, the cost of the vaccine was the least cited barrier toward vaccine uptake. This suggests the willingness of the public to take the vaccine if they were aware of its availability. In addition, it stresses the need for governmental efforts to educate the public about the availability of the vaccine in the country. In addition to unawareness of vaccine availability, doubts regarding the safety of the vaccine and not considering influenza as a threat were other highly cited reasons for abstinence from vaccination. In agreement with these results, similar studies from Jordan and the United States showed that low perceived risk of influenza and concerns regarding the safety and efficacy of the vaccine are leading factors for rejecting vaccination (30, 39). Additional challenges in Yemen such as political conflict, personal safety, food security, weak infrastructure, collapsed healthcare system, and lack of awareness campaigns on the importance and availability of influenza vaccine are all potential barriers to vaccination (67, 68).

When assessing factors that will encourage the participants to receive the influenza vaccine in the future, most participants expressed their willingness to take the vaccine if it was recommended by the physicians, was sufficiently validated for safety and efficacy, and was advocated and offered free of charge by the government. Similarly, in a recent study from Jordan, most participants were willing to get vaccinated if the influenza vaccine was recommended by physicians, was safe and effective, and was provided free by the government (39).

Overall, the study at hand is an important first step to inform researchers and decision-makers in the country of the current public awareness toward influenza and its vaccine, including the low vaccination coverage. Intervention studies, utilizing motivators and barriers toward vaccine uptake reported in the study, are likely to follow. Implementing equitable access to information about influenza and its vaccine in the country is possible using the available multimedia in the country, for instance, utilizing short SMS mobile phone messages to inform the public of information about influenza and the availability of the vaccine. On the other hand, equitable access to influenza vaccine would seem more challenging given the current conditions in the country. The latest World Bank data show that most of the Yemeni population (~75%) live below the poverty line. Therefore, one important step toward implementing equitable vaccine access would be to offer it free of charge, for instance as part of the national immunization program.

## 5. Limitations

The current study comes with some limitations that can be mitigated through future research. One such limitation is that the current study inquired about “ever” receiving the flu vaccine. Future research can explore vaccine uptake in each year/duration, which will provide valuable insight into the effect of the ongoing conflict on vaccine uptake and the change in the annual vaccination coverage over time. Moreover, although our study recruited participants from different areas of different cities, our sampling method, by definition, is convenience sampling and the ability to generalize from convenience sampling remains limited compared to random sampling. Randomly sampling the population would have been really challenging given the current state of war in the country. In addition, even though our study did not assess the economic status of the participants, it still can be assumed from the health insurance statistics that most participants are from the low-to-middle class as only 25% of participants reported having health insurance, which is not mandatory in the country and is not covered by government or private employers, and therefore only 25% would seem to afford to have health insurance and, probably, the flu vaccine as well. However, our univariate and multivariate analyses did not reveal having health insurance as a predictor for vaccine uptake. As such, the low vaccine uptake reported in the current study would not seem to be attributed to the less wealthy participants but rather to other factors such as the lack of awareness of the availability of the vaccine which has been cited as the main barrier to vaccine uptake by the participants. Another limitation is that the assessment of influenza vaccine uptake was based on the self-reporting recall of the participants rather than reviewing medical records.

## 6. Conclusion

Critical gaps in knowledge of influenza were identified among the public in Yemen. The study revealed a low vaccine uptake in the country and identified major determinants of vaccine acceptance and rejection. Optimizing vaccine acceptance and coverage can be achieved by collaboration between the healthcare sector and governmental authorities. Efforts ensuring the free-of-charge provision of the vaccine will assess in establishing equitable vaccine access. In addition, implementing education programs utilizing different audiovisual platforms is recommended to enhance positive attitudes toward influenza vaccine, raise awareness toward vaccine availability, consolidate the public's trust in the safety of the vaccine, and promote the vaccine among high-risk groups in the community who are in critical need of the vaccine.

## Data availability statement

The original contributions presented in the study are included in the article/supplementary material, further inquiries can be directed to the corresponding author.

## Ethics statement

The studies involving human participants were reviewed and approved by Yemen University (Ref #: ERC/2018/123). Written informed consent for participation was not required for this study in accordance with the national legislation and the institutional requirements.

## Author contributions

WA: conceptualization, study design, data interpretation, original draft preparation, project administration, reviewing, and editing. RA: data analysis, data interpretation, original draft preparation, reviewing, and editing. AH: data acquisition, data interpretation, original draft preparation, reviewing, and editing. AA and YA-A: data acquisition, reviewing, and editing. EA-r: study design, reviewing, and editing. MZ: data interpretation, reviewing, and editing. SA-J: conceptualization, study design, data acquisition, data interpretation, original draft preparation, and project administration. All authors contributed to the article and approved the submitted version.
